# (−)-Oleocanthal Prevents Breast Cancer Locoregional Recurrence After Primary Tumor Surgical Excision and Neoadjuvant Targeted Therapy in Orthotopic Nude Mouse Models

**DOI:** 10.3390/cancers11050637

**Published:** 2019-05-08

**Authors:** Abu Bakar Siddique, Nehad M. Ayoub, Afsana Tajmim, Sharon A. Meyer, Ronald A. Hill, Khalid A. El Sayed

**Affiliations:** 1 School of Basic Pharmaceutical and Toxicological Sciences, College of Pharmacy, University of Louisiana at Monroe, 1800 Bienville Drive, Monroe, LA 71201, USA; siddiqab@warhawks.ulm.edu (A.B.S.); tajmima@warhawks.ulm.edu (A.T.); meyer@ulm.edu (S.A.M.); rhill@ulm.edu (R.A.H.); 2 Department of Clinical Pharmacy, Faculty of Pharmacy, Jordan University of Science and Technology, Irbid 22110, Jordan; nmayoub@just.edu.jo

**Keywords:** breast cancer, extra-virgin olive oil, HER2, lapatinib, MET, neoadjuvant, oleocanthal, surgical excision, recurrence

## Abstract

Breast cancer (BC) recurrence represents a challenge for survivors who have had their primary tumors surgically excised, and/or have completed radiation, neoadjuvant, or adjuvant therapeutic regimens. Current BC treatments mostly lack the ability to reduce the risk of disease recurrence. About 70% of BC patients will subsequently suffer disease relapse, manifesting as local, regional, or distant tumor recurrence, which clearly underscores the urgent need to discover novel recurrence inhibitors. (−)-Oleocanthal (OC) is a natural phenolic, found so far exclusively in extra-virgin olive oil (EVOO). OC exerts documented bioactivities against diverse cancer types, inflammation, and neurodegenerative diseases. Herein we report the novel activity of daily oral treatment with OC (10 mg/kg) in preventing BC locoregional recurrence in a nude mouse xenograft model generated by orthotopic inoculation with BT-474 cells as a luminal type B model. We further report inhibition of tumor recurrence by OC after completion of a lapatinib neoadjuvant regimen. However, in a recurrence model of triple-negative breast cancer (TNBC), OC treatment (10 mg/kg) did not effectively prevent tumor recurrence, but rather, was seen to significantly reduce the growth of recurrent tumors as compared to vehicle control-treated animals. Inhibition of tumor recurrence was associated with significant serum level reductions of the human BC recurrence marker CA 15-3 at the study end in animals treated with OC. OC treatment upregulated the expression of the epithelial marker E-cadherin and downregulated the levels of the mesenchymal marker vimentin in recurrent tumors vs. untreated control animals. OC treatment also reduced the activation of MET and HER2 receptors, as indicated by reduced phosphorylation levels of these proteins in recurrent tumors vs. controls. Collectively, the results of our studies provide the first evidence for suppression of BC tumor recurrence by oral OC treatment in an animal model for such recurrence, and furthermore, highlight favorable prospects for this natural product to emerge as a first-in-class BC recurrence inhibitor.

## 1. Introduction

Breast cancer (BC) is the most commonly diagnosed cancer and the second-leading cause of cancer-related death in women worldwide [1,2]. Globally, two million new BC cases are expected to be diagnosed in 2019, with an estimated 627,000 women anticipated to die from BC complications [1,2]. Predictions are that in 2019 alone, more than 268,000 US women will be diagnosed with BC [3,4], and that BC will claim the lives of more than 41,000 US women [3,4]. BC is a heterogeneous disease that is classified into four major molecular subtypes: luminal A, luminal B, human epidermal growth factor receptor (HER2)-overexpressing, and basal-like, based on clinical, histopathological, and microarray criteria [3,4,5]. Mortality is mainly attributed to metastatic or recurrent disease [5].

Excision surgery is a mainstay modality for treatment of BC. Surgical excision of a primary tumor mass is indicated for tumors confined to breast tissue among patients with early-stage disease or even locally advanced cases. Surgical resection of BC greatly improves cure rates among such patients, and minimizes the potential for metastasis to distant sites [6,7]. Neoadjuvant and adjuvant therapeutic agents in current use have limited efficacy in preventing BC recurrence and/or metastasis. Interestingly, neoadjuvant therapy has been shown to increase the rate of locoregional recurrence [8]. Recently, neoadjuvant taxanes and anthracyclines have been shown to induce extracellular vesicles with enhanced pro-metastatic capacity in BC models [9]. Despite progress in endocrine and targeted therapies and the consequent improvements in survival rates among BC patients, these modalities are largely ineffective in recurrence prevention. Furthermore, development of resistance to endocrine therapies, targeted therapies, and chemotherapeutic agents is increasing, which further reduces the chances of recurrence-free survival [7,10,11,12]. Resistance can develop via dysregulation of alternative growth factor receptors, including epidermal growth factor receptor (EGFR) and HER2, as well as any of a number of oncogenic signaling pathways or a combination thereof, including the PI3K/AKT and Ras/Raf/MAPK pathways, which may result in estrogen receptor (ER) ligand-independent activation [11,12,13]. In addition to resistance, the high cost of some targeted treatments, along with their associated adverse effects, necessitates the search for novel BC therapeutics with tolerable adverse effect profiles and greater potential for preventing tumor recurrence.

A wealth of data documents the reduced risk of Mediterranean populations to certain chronic diseases typically emerging later in life, some associated with slow devolvement from healthy status over many years, including atherosclerosis, cardiovascular disease, and particular types of cancer, in addition to extended life expectancy as compared to populations of other geographical regions [14,15,16,17,18]. These favorable health outcomes have been widely attributed—based on much corroborating epidemiological evidence—to the regular consumption of extra-virgin olive oil (EVOO), which is a major component of the Mediterranean diet [12,13,14,15,16]. EVOO contains a number of minor bioactive phenolic compounds, including simple phenols, lignans, and secoiridoids, in addition to its relatively high unsaturated fatty acid content [14,15,16].

(−)-Oleocanthal (OC) was first isolated by the Montedoro group from EVOO, and later was identified by the Beauchamp group as the potent nonsteroidal anti-inflammatory EVOO constituent having ibuprofen-like cyclooxygenase inhibitory activity [19,20]. OC exhibited antioxidant and neuroprotective activities, which conferred beneficial effects in cellular and animal models for Alzheimer’s disease [21,22,23]. In addition, OC exerts anti-inflammatory activity mediated by inhibition of IL-6 expression, and of 5-lipoxygenase secretion [21,23,24]. Effects of OC indicative of anticancer potential have been seen in various cell culture and animal models. These effects are so far known to be mediated, at least in part, via inhibition of mesenchymal-epithelial transition factor (MET) receptor tyrosine kinase and its downstream signaling pathways [25,26], suppression of phosphorylated mechanistic target of rapamycin (mTOR) levels [27], suppression of cyclooxygenase-2 (COX-2) expression [28], and inhibition of signal transducer and activator of transcription 3 (STAT3) [29] in models for various cancer types.

Consistent with its in-vitro effects, OC markedly reduced growth of tumors and suppressed hepatocellular carcinoma metastasis to lungs in animal models [29]. In support of OC’s potential for health promotion and cancer suppression vs. deleterious effects (i.e., therapeutic index), OC was not seen to adversely affect the viability and growth of non-tumorigenic human mammary epithelial cells [26]. Earlier, our group validated the in-vitro and in-vivo efficacy of OC in favorably modulating ER expression and function in hormone-dependent BC models, and synergy with the selective ER modulator tamoxifen [30]. Recently, we also showed that OC significantly synergized with lapatinib in both in-vitro and in-vivo (animal) model systems for hormone-dependent BC [31]. Orally dosed OC was also recently shown to significantly suppress the growth of triple-negative BC (TNBC) in a nude mouse orthotopic xenograft model [32]. Collectively, these results, in the context of other prior investigations of olive oil’s multifaceted beneficial effects, along with considerable epidemiological evidence for EVOO consumption as being a linchpin component accounting for the cancer-suppressing effects of diet in Mediterranean populations, compellingly support the hypothesis that the phenolic constituent OC might be at least a key constituent of EVOO responsible for conferring these highly desirable benefits.

Taking into consideration the highly adverse impact that tumor recurrence imposes on patient survival, in conjunction with the in-vivo anticancer activities convincingly seen in a number of studies of OC, we turned our attention to investigating, in a small array of diverse cancer cell types and experimental paradigms, the potential of OC treatment for preventing recurrence. Minimal research efforts have been expended towards the discovery of inhibitors of recurrence and metastasis, in part because clinical trials on these treatment endpoints may be not financially feasible or rewarding, given that such trials are extremely lengthy and need large numbers of patients for sound interpretability and validity [33,34,35]. Therefore, it is important at this juncture to seek natural product-based recurrence inhibitors, partly because the time entailed for their translational development for use as dietary supplements by BC patients should be shorter than for medicinal agents classified as drugs. Also, easily manageable BC recurrence inhibition animal models with good relevance to humans will be needed for facilitating recurrence inhibitor discovery and aspects of pre-clinical development.

The potential of OC as an inhibitor of BC recurrence might have a basis in its potent inhibition of MET receptor function and its associated downstream signaling pathways. The hepatocyte growth factor (HGF)/MET pathway plays a role that appears could be essential in at least some BC recurrence, a contention further supported with evidence obtained by MET immunostaining in primary human donor BC samples, especially when reconstructive fat grafts are used with surgical resection, [33]. Residual surviving cancer cells have been shown to repopulate, incepting new tumors after surgical excision or during a gap of chemotherapy cycle periods [34,35]. MET amplification is one molecular mechanism leading to activation of quiescent tumor cells, which can thence cause repopulation of cancer cells and subsequent tumor recurrence and relapse [7,33,34].

In this study, we established a new BC recurrence animal model, based on orthotopic inoculation of tumor cells into nude mice mammary fat pads. Initially, the cells create a primary tumor, which is then surgically excised, followed by treatment with either test compound or vehicle control for several weeks, in all instances comparing with results obtained for the same regimen of vehicle control. Locoregional recurrence is then assessed by monitoring the development of newly appearing tumors at the site of surgical excision, which are further measured for volume and ultimately excised at the end of each study. OC treatments were administered in this study either alone, or after completing presurgical (neoadjuvant) regimens with the dual EGFR–HER2 inhibitor lapatinib (LP), in a test system intended to model HER2-positive BC. The first objective was to assess the ability of OC as the sole treatment agent to prevent HER2/ER-positive BC and TNBC locoregional recurrence after primary tumor surgical excision. The second objective was to investigate whether OC would be able to prevent recurrence, following resection of primary tumors generated with HER2/ER-positive BT-474 cells, after completion of a neoadjuvant LP regimen.

## 2. Results

### 2.1. Oleocanthal Inhibits Locoregional Recurrence of BT-474 Tumors in a Nude Mouse Orthotopic Xenograft Model

**Figure 1:**
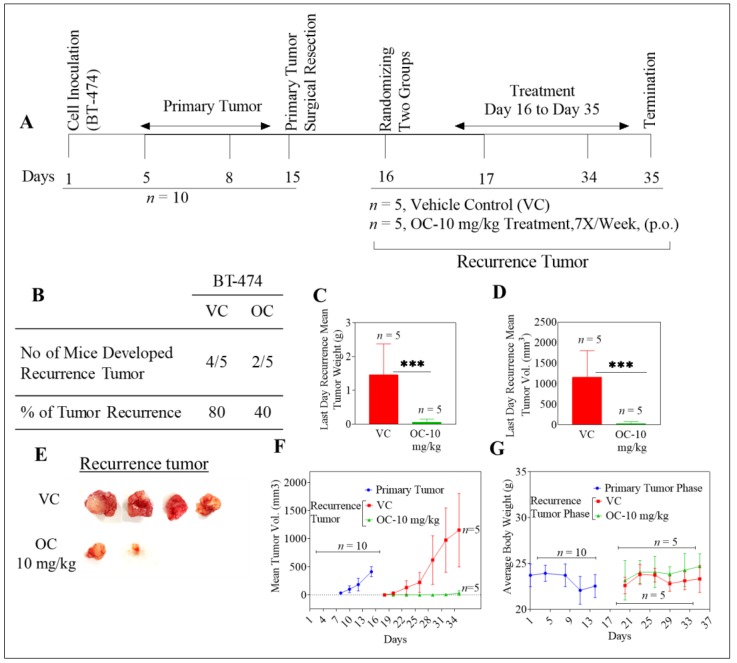
Oleocanthal treatment inhibited recurrence of BT-474 tumors in a xenograft orthotopic nude mice model. (**A**) Overview of the experimental design. (**B**) Incidence of BC recurrence in each experimental group. (**C**) Mean weight comparison (treated vs. control) for recurring tumors at the end of the experiment. Error bars indicate SD. *** *p* < 0.001 value represents the statistical significance as compared to vehicle-treated control group. (**D**) Mean volume comparison (treated vs. control) for recurring tumors at the end of the experiment. Error bars indicate SD. (**E**) Representative recurrent BT-474 breast tumors for each experimental group. (**F**) Mean tumor volumes for primary and recurrence phases over the treatment period. Data points represent the mean tumor volume, error bars indicate SD, for each experimental group. (**G**) Body weights (mean ± SD) of animals in each group during the primary and recurrence phases of the experiment.

BT-474 BC cells represent the luminal B subtype, which is positive for the expression of hormone receptors and HER2 [36]. After inoculation with BT-474 cells, all mice (*n* = 10) developed primary breast tumors. These tumors were surgically excised once the average tumor volume reached 400 mm^3^. The H&E stained images of the excised primary tumors showed distinct outer peripheral margin of normal cells encrusting the primary tumor core tissues, confirming effective surgical removal of primary tumors (Figure S1A). All mice were healthy after surgery and therefore could be randomly parsed into two groups, *n* = 5 each. One group was treated with vehicle control and the other group with OC administered orally by gavage at 10 mg/kg, as detailed later in Materials & Methods. At the end of the study, 4 out of 5 mice developed tumor recurrence (80%) in the vehicle control group, while only 2 out of 5 mice developed recurrent tumors (40%) in the OC-treated group (Figure 1A,B). The mean tumor weight of vehicle-treated and OC-treated groups was 1.5 ± 0.9 g and 0.2 ± 0.1 g, respectively (Figure 1C). The mean tumor volume was 1152.9 ± 652.8 mm^3^ and 53 ± 47.4 mm^3^ for vehicle control and OC-treated mice, respectively (Figure 1D–F). Tumor growth inhibition (TGI; see Methods section) for OC was around 95% for recurrent tumors. Mean body weight for animals in vehicle-control and OC-treated groups did not significantly differ throughout the study duration (Figure 1G). All animals completed the whole experiment course until the study was terminated. Weights of body organs in animals from both vehicle-control and OC-treated groups were recorded at the study end, with no significant statistical difference observed between the two groups (Table S1).

Histopathology of the primary and recurrent tumor samples showed that several mitotic features were evident in recurrent BT-474 tumors in mice treated with vehicle control, including one tumor with abnormal morphology (Figure S1A,B). Recurrent tumors of OC–treated mice with resected BT-474 cell-generated primary tumors showed notable cytolethality, with extensive cell disintegration leaving granular and vacuolar debris and few intact cells remained (Figure S1B).

### 2.2. Oleocanthal Reduces Growth of Recurrent MDA-MB-231 Tumors in a Nude Mouse Orthotopic Xenograft Model

**Figure 2:**
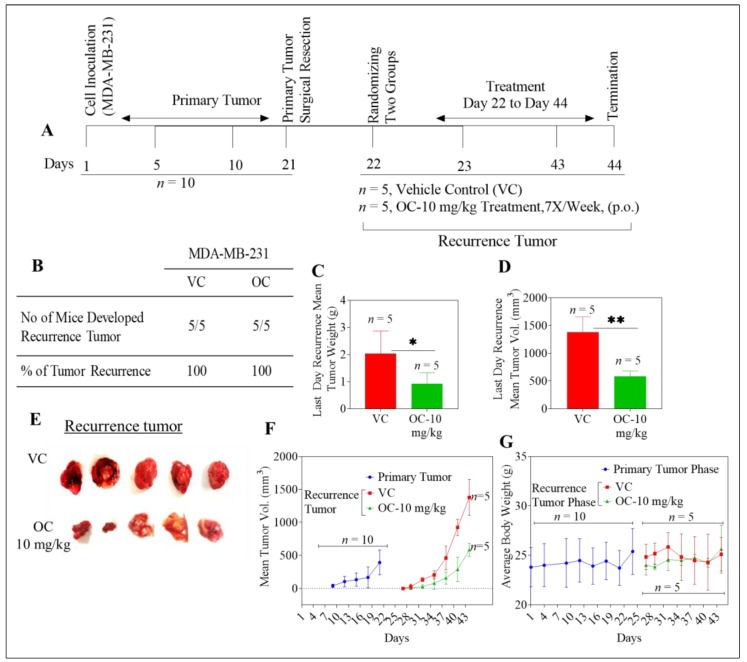
Oleocanthal reduced growth of recurrent MDA-MB-231 tumors in a xenograft orthotopic nude mice model. (**A**) Overview of the experimental design. (**B**) Incidence of TNBC recurrence in each experimental group. (**C**) Mean weights (treated vs. control) for recurring tumors at the end of the experiment. Error bars indicate SD. * *p* < 0.05 for statistical significance comparing to vehicle-treated control group. (**D**) Mean volume comparison (treated vs. control) for recurring tumors at the end of the experiment. Error bars indicate SD. ** *p* < 0.01 for statistical significance. (**E**) Representative recurrent MDA-MD-231 breast tumors for each experimental group. (**F**) Mean tumor volumes for primary and recurrence phases over the treatment period. Points represent the mean tumor volume, error bars the SD, for each experimental group. (**G**) Body weight monitoring data over the experiment course. Points represent the mean body weight for animals in each group at each weighing. Error bars indicate SD.

Cells of the MDA-MB-231 BC line lack pronounced expression of hormone receptors and of HER2, and are thus a valid choice for generating models of TNBC [36]. After inoculation with cells of this line, all animals (*n* = 10) developed primary breast tumors, which were then surgically excised once the average tumor volume reached 400 mm^3^. Histopathological examination of the H&E stained images of the excised primary tumors clearly showed distinct outlining with a normal tissue marginal layer at the periphery of the primary tumor, consistent with effective (i.e., complete and clean) surgical removal (Figure S1A). All mice were healthy after the surgery, and therefore were randomized into two groups (*n* = 5), following which oral treatments with either vehicle or with OC at 10 mg/kg were initiated, as earlier described. For primary tumors generated with this TNBC model cell line, all mice (100%) in both vehicle control and OC treatment groups developed breast tumors by the end of the study (Figure 2A,B). The mean weight for tumors at the end of the recurrence phase was 2.03 ± 0.8 g and 0.92 ± 0.4 g in vehicle control and OC-treated groups, respectively (Figure 2C). In addition, mean tumor volumes were 1380.1 ± 274.1 mm^3^ and 583.6 ± 95.9 mm^3^ for the vehicle control and OC-treated mice groups, respectively (Figure 2D–F). Though OC did not inhibit tumor recurrence in this model of TNBC (Figure 2B), OC significantly suppressed tumor growth, by 58%, as compared to control-treated animals. Body weight means for vehicle-control and OC-treated groups were not significantly different throughout the duration of experiment (Figure 2G). No statistically significant differences were seen for mean organ weights between the two treatments groups at the end of the study (Table S1).

In histopathological examinations of MDA-MB-231 recurrent tumor samples, mitotic features were evident for tumors in mice treated with vehicle alone after primary tumor excision. In contrast, tumors of OC-treated animals exhibited various dysplasias, indicative of cytotoxicity, including marginalized chromatin, cytoplasmic eosinophilia with indistinct basophilic structures, and clear pericellular space characteristic of apoptosis (Figure S1B). Giant cells were also evident, with emperipolesis (Figure S1B).

### 2.3. Oleocanthal Inhibits Locoregional Recurrence of BT-474 Tumors in a Nude Mouse Orthotopic Xenograft Model After Neoadjuvant LP Treatment

**Figure 3:**
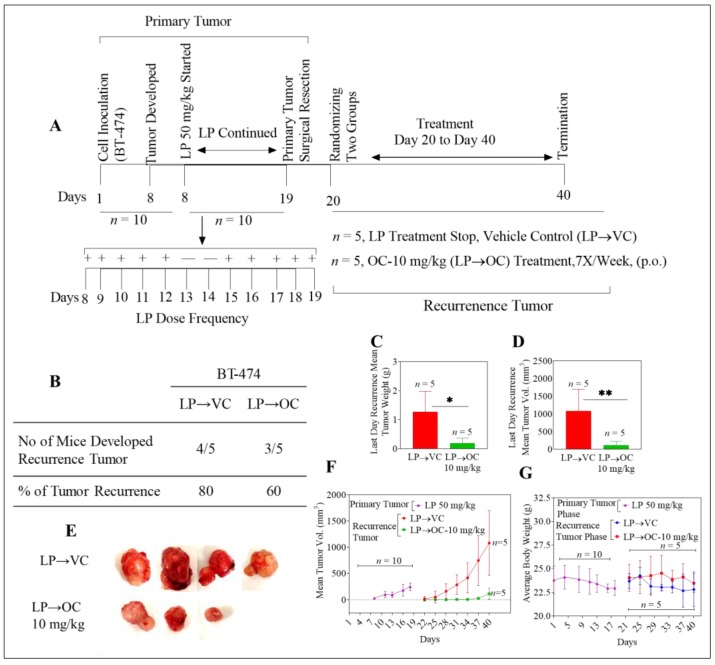
Oleocanthal inhibits recurrence of BT-474 tumors in a xenograft orthotopic nude mouse model after neoadjuvant LP treatment. (**A**) Overview of the experiment layout. (**B**) Incidence of tumor recurrence in each experimental group. (**C**) Mean tumor weight comparison (treated vs. control) at the end of the experiment. Error bars indicate SD. * *p* < 0.05 when comparing treated to vehicle control groups. (**D**) Mean tumor volume at the end of experiment for tumor recurrence, comparing vehicle control (LP→VC) and OC-treated (LP→OC) groups. Error bars indicate SD. ** *p* < 0.01. (**E**) Representative recurrence tumors from each experimental group. (**F**) Mean tumor volumes for primary and recurrence phases over the treatment period. Points represent the mean tumor volume, error bars indicate SD for each experimental group. (**G**) Mean body weights of animals in each group during the primary and recurrence phases of the experiment. Error bars indicate SD.

Lapatinib (LP) is a dual HER2/EGFR kinase inhibitor which is currently an approved therapeutic option as a second-line treatment for HER2-positive metastatic BC patients [37]. After inoculation with BT-474 cancer cells, all mice (*n* = 10) developed primary breast tumors. LP neoadjuvant treatment (50 mg/kg) was initiated when tumors were at an average volume of 30 mm^3^, continuing until day 19. Primary breast tumors were surgically excised once the average tumor volume reached 400 mm^3^, or upon completion of the LP treatment regimen, whichever was earlier. H&E stained images of excised primary tumors in all cases showed a distinct margin of normal cells outlining the primary tumor tissue, confirming clean primary tumor removal (Figure S1A). All mice were healthy after the surgery and therefore could be randomly parsed into two groups (*n* = 5). One group started oral treatment with vehicle control (LP→VC). The other group started oral OC treatment at 10 mg/kg (LP→OC) as detailed later in Materials & Methods. At the end of the study, 4 out of 5 mice (80%) in the vehicle control group developed recurrent tumors, while only 3 out of 5 mice (60%) developed recurrent tumors in the OC-treated group (Figure 3A,B). Tumor weight means for vehicle-treated and OC-treated groups were 1.26 ± 0.7 g and 0.19 ± 0.2 g, respectively (Figure 3C). Mean tumor volumes were 1080.6 ± 615.8 mm^3^ and 116.6 ± 110.4 mm^3^ for vehicle control and OC-treated mice, respectively (Figure 3D–F). The OC-treated group (LP→OC) showed 89% TGI compared to the vehicle-treated control group (LP→VC). No statistically significant differences in body weight averages were discerned when comparing the control and OC-treated groups at any given time point over the study duration (Figure 3G). Mean organ weights did not statistically differ between control and treatment groups (Table S2).

Histopathological examination of primary tumor samples from BT-474 inoculated animals treated with LP revealed the presence of binucleated cells, fibrotic tracks, vacuolization, and reduced mitosis due to the drug effects (Figure S1A). Viable BT-474 tumor cells were present in both primary and recurrent tumors. Animals in the OC-treated group showed more regions of evident coagulative necrosis (Figure S1B).

Brain, lung, and liver collected from all animals were sectioned, H&E stained, and histopathologically examined for micrometastasis. None of the organs showed tumor micrometastasis.

### 2.4. Oleocanthal Reduced Human CA 15-3 Levels in Serum of BT-474 and MDA-MB-231 Orthotopically Xenografted Nude Mice

Blood was collected from all mice immediately after sacrifice at the experiment end, and serum samples were prepared. Cancer antigen 15-3 (CA 15-3) is a marker of BC recurrence that was measured in sera using the Abnova solid-phase enzyme-linked immunosorbent assay (ELISA, Figure S2). Serum levels of CA 15-3 averaged significantly higher in control animals as compared to OC-treated animals for both luminal and TNBC models of tumor recurrence (Table 1). In animals having BT-474 generated tumors, mean serum levels of CA 15-3 in vehicle control group and OC-treated mice were 1.9 ± 0.9 and 0.4 ± 0.4 U/mL, respectively (Table 1). In animals with MDA-MB-231 generated tumors, mean CA 15-3 levels for the vehicle control group animals and the OC-treated mice were 2.1 ± 0.8 and 0.4 ± 0.1 U/mL, respectively. In LP neoadjuvant treated animals, mean levels of CA 15-3 in vehicle control (LP→VC) and OC-treated (LP→OC) groups were 1.6 ± 1.0 and 0.4 ± 0.1 U/mL, respectively (Table 1).

### 2.5. Effect of Oleocanthal Treatment on EMT Markers, Total and Active MET and HER2 RTKs in BC Recurrence Models

**Figure 4:**
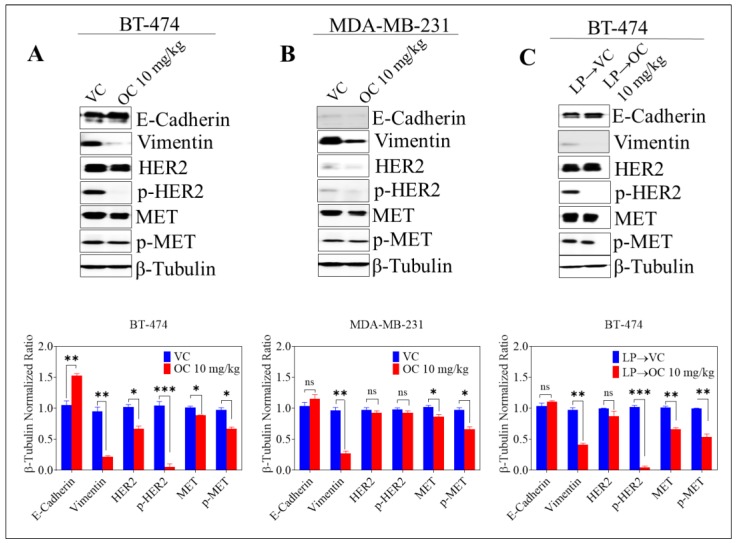
Effect of oleocanthal treatment on E-cadherin, vimentin, total and active MET and HER2 RTKs in BC recurrence models. Top panels include representative Western blots for the indicated markers, and bottom panels represent the results of densitometric analysis performed on all blots. (**A**) Expression of the indicated markers in recurrent tumors from the BT-474 recurrence model animals in the different experimental groups, resected at the end of each experimental treatment protocol, (**B**) for tumors resected from MDA-MB-231 recurrence model animals, (**C**) for tumors resected from BT-474 recurrence model animals after neoadjuvant LP. Scanning densitometry was obtained for all blots, carried out in triplicates, and the integrated optical density of each band was normalized with the corresponding density found for β-tubulin in the same blot, results shown in the bar graphs under their respective Western blot images. Vertical bars in the graph indicate the normalized integrated optical density of bands visualized in each lane. * *p* < 0.05, ** *p* < 0.01 and *** *p* < 0.001 compared to respective vehicle-treated control group.

OC treatment increased E-cadherin expression and decreased vimentin levels in BT-474 generated tumors, whether treated with neoadjuvant LP or not, as compared to vehicle control (Figure 4A,C). In addition, OC treatment significantly suppressed HER2 activation, again with or without neoadjuvant LP, as indicated by greatly reduced levels of phosphorylated HER2 (p-HER2) in OC-treated animals as compared to vehicle-controls (VC) (Figure 4A,C). In MDA-MB-231 cell generated tumors, OC treatment once again significantly reduced vimentin expression; however, it was not effective in renormalizing expression of E-cadherin in these tumors. Due to minimal expression of HER2 in MDA-MB-231 cells, the effect of OC treatment on HER2 activation in MDA-MB-231 tumors was not remarkable (Figure 4B). In BT-474 generated tumors, OC modestly reduced total and activated MET levels in recurrent tumors, with or without prior exposure to neoadjuvant LP treatment, as compared to VC groups (Figure 4A,C, bar charts at bottom), and the same was true in MDA-MB-231 generated recurrent tumors (Figure 4B, bottom).

## 3. Discussion

In-vivo anticancer activities of OC have been seen in multiple studies. OC treatment markedly inhibited the growth of xenograft tumors representing different molecular subtypes of BC [26]. For example, Akl et al. (2014) demonstrated that intraperitoneal (ip) treatment with OC markedly reduced tumor formation and growth of TNBC-model xenogenic orthotopic tumors in nude mice [26]. Further, OC suppressed the growth of BT-474 generated tumors in an orthotopic mouse model of luminal BC [30]. Recently, OC was seen to significantly synergize with LP in inhibiting the growth of BT-474 generated tumors, and to effectively suppress the growth of TNBC-model tumors in animals when administered orally [31,32]. Despite the accruing evidence for OC as an inhibitor of BC growth in-vivo in such models, the potential of OC treatment for combating BC recurrence has not been previously investigated. Findings in this study showed, for the first time, that OC can significantly suppress the development of new tumors and prevent BC locoregional recurrence, though our results also suggest that scope may be limited according to cancer type.

Surgery is the mainstay treatment modality for patients with early stage BC [38]. Over the past few decades, the surgical approach for BC treatment has undergone a major shift from traditional modified radical mastectomy to applying breast conservative surgery (BCS) with radiotherapy. Despite advantages and self-evident attractions of BCS over mastectomy, however, the former has been associated with a greater incidence of locoregional recurrence [38]. Recurrence, as a leading cause of mortality among patients, thus represents a major therapeutic challenge to oncologists [33]. These facts warrant further investigations into ways to reduce the risk of BC relapse, and thereby improve longer-term patient survival rates. Based on the systemic disease concept of BC, surgical excision of primary breast tumors does not preclude the potential of distant disease [38]. Clinically, local recurrence is defined as recurrence of BC in the original tumor bed or field of mastectomy in patients [38]. Regional recurrence involves the presence of metastatic disease in the ipsilateral axilla or supraclavicular lymph nodes alone, or in combination with the involvement of the ipsilateral breast [38]. Predictors of increased risk of both locoregional and distant recurrence after surgery in BC include tumor size, nodal status, presence of lymphovascular invasion, and high histologic grade [38,39]. Importantly, the pattern and rate of recurrence were associated with BC intrinsic molecular subtypes, independent of tumor clinicopathologic characteristics [40,41]. Distant disease recurrence in HER2-positive and basal tumors was generally associated with a greater tendency toward systemic disease than luminal types [41]. HER2-enriched tumors also lend to higher rates of brain, liver and lung metastases compared with luminal A tumors [35,40,42]. TNBC is associated with more visceral metastases, and considerably increased risk of lung and brain involvement, as preferred sites of recurrent disease [34,35,41,43]. Given this body of findings, in the current study we focused on evaluating outcomes for local and regional recurrence using an orthotopic xenograft nude mouse paradigm, with two arms of experimental models representing HER2-positive BC and TNBC.

Pronounced amplification of the expression of the HER2 protein is associated with cancer cell proliferation, migration, and invasiveness [44]. Clinically, HER2 overexpression is associated with decreased rates of disease-free and overall survival (OS) in BC patients [45]. Interestingly, the site and pattern of distant recurrences in patients whose tumors are HER2-positive may be further dependent on hormone receptor status [45,46]. Hess and Esteva found that the effect of HER2 status on the development of brain metastasis is stronger in patients with ER-positive cancers as compared to patients with ER-negative tumors [45]. The introduction of targeted anti-HER2 therapies has improved outcomes in a significant fraction of BC patients with HER2-positive disease; however, 15% of patients will still develop distant metastasis/recurrence despite having undergone current consensus state-of-the-art local and systemic treatment protocols [45].

In the studies reported herein, OC treatment inhibited local recurrence of BT-474 generated orthotopic xenograft tumors. BT-474 cells are known to richly express both HER2 and ER. In this model, OC prevented tumor recurrence in 50% of treated animals as compared to the vehicle control group. In addition, treatment with OC suppressed growth of recurrent tumors that did appear. OC also showed activity in inhibiting tumor recurrence in animals treated with neoadjuvant LP before surgical removal of primary tumors. BT-474 cells richly express MET, and overexpress HER2, both of which have been shown to be downregulated in response to OC, both in-vitro and in-vivo [30,36]. Findings from this study highlight the potential of OC treatment to prevent tumor recurrence after surgery and targeted (cancer-type-guided) neoadjuvant treatment in BC patients with the HER2/ER-positive phenotypic subtype. In this regard, and based on our preliminary findings, OC may have the potential to be an appealing option for longer-term prevention of BC recurrence, taking into consideration its remarkable selectivity to targeting cancer cells versus non-tumorigenic mammary epithelial cells [26,29,30,31] as well as lack of apparent systemic toxicity (corroborated herein by lack of gross alterations in body and organ weights in animals over the course of the experiment).

The hepatocyte growth factor (HGF) and its receptor tyrosine kinase, MET, play key roles in cancer development and progression as a result of their involvement in generating mitogenic, motogenic, and angiogenic activities [47]. Aberrant HGF/MET activation and/or expression result in an aggressive phenotype and is associated with tumor progression, metastatic potential, and poor survival in BC patients [48,49]. Increased levels of total and phosphorylated MET have been reported in BC patients among various molecularly subtyped cancers [47]. Raghav et al. showed that total and phosphorylated MET levels are significant prognostic indicators for relapse-free survival (RFS) and OS in BC patients [47]. In this survival analysis, high MET expression portended poor prognosis for patients with ER-positive BC, and high MET phosphorylation levels were associated with poor prognosis for HER2-positive tumors [47].

TNBC cells from patients do not overexpress HER2 or exhibit enriched hormone receptor expression, and these patients thus are not valid candidates for endocrine treatment or HER2-targeted therapy. So far, chemotherapy is the mainstay systemic treatment for this subtype of BC. In such settings, MET may represent an important potential therapeutic target in TNBC. In line with this assertion, Zagouri et al. showed that RFS and OS were shorter in TNBC patients with high MET expression than those with low tumor levels of this RTK [50]. Results from our study showed that OC treatment did not prevent recurrence in the TNBC model. All animals in both control and OC-treated groups developed recurrent tumors after surgical resection of their primary MDA-MB-231 tumors. Nevertheless, oral OC treatment was effective in suppressing the growth rates of new tumors, as indicated by significantly reduced tumor volumes and TGI of 58% compared to vehicle control animals. The lack of recurrence-preventive effects of OC for MDA-MB-231 generated orthotopic xenograft tumors can be rationalized in the context of the biology of TNBC. In general, TNBC is associated with a worse prognosis compared to other BC subtypes, which could be explained phenotypically, at least in-part, by the aggressive nature of these tumors, in terms of their high invasive potential [41]. In a recent study, Yano et al. showed that locally recurrent tumors from MDA-MB-231 generated primary tumors grew more rapidly than did parental MDA-MB-231 primary tumors after surgical resection in orthotopic nude mouse models [40]. The study also provided evidence for extensive lymph-node metastases after primary tumor resection compared to parental MDA-MB-231 generated primary tumors [51]. Wright et al. found triple-negative receptor status to be a risk factor for locoregional recurrence among BC patients who received neoadjuvant therapy, underwent mastectomy, and also had post-mastectomy radiation [52].

Biomarkers have diagnostic and predictive values in cancer management. Blood biomarkers are especially important for early diagnosis of cancer and future prediction of therapeutic responses [53]. Cancer antigen 15-3 (CA 15-3) is an important tumor marker for monitoring post-operative risk of recurrence and metastasis in cancer patients [54]. Blood levels of CA 15-3 are elevated in more than 96% of patients with local and systemic cancer, further validating the use of CA 15-3 to predict early tumor recurrence. Further, CA 15-3 is more sensitive than carcinoembryonic antigen (CEA) for the early detection of BC recurrence [55]. In this study, OC treatment was seen to be associated with reduced CA 15-3 levels in the sera of mice treated with OC orally, as compared with vehicle control, in all three models of BC recurrence.

Multiple pathways are involved in mediating recurrence of BC in different patients [56]. In this regard, epithelial-to-mesenchymal transition (EMT) has attracted increasing attention to predict BC recurrence potential [34]. MET is a key regulator of EMT, and sustained activation of the HGF/MET pathway results in downregulation of the expression of the epithelial markers E-cadherin and cytokeratins 8/18, along with upregulation of the mesenchymal protein vimentin [57,58]. In earlier studies, recurrent tumors reportedly exhibited low expression of E-cadherin and high expression of vimentin [55]. Cancer cells undergoing EMT eventually lose their epithelial cell characteristics in acquiring a mesenchymal phenotype, gaining migratory and invasive properties [57]. Khoury et al. demonstrated that HGF/MET downstream signaling nefariously synergizes with cellular consequences of HER2 overexpression to enhance the malignant phenotype through breakdown of cell–cell junctions and promotion of cell invasion [59]. In line with this, Previdi et al. showed that HGF/MET stabilizes β-catenin in a bone metastatic clone derived from MDA-MB-231 cells, which further promoted inception and progression of bone metastases in BC [60]. In the experimental paradigms and protocols of our studies reported herein, we documented that OC treatment stabilized E-cadherin and concomitantly reduced vimentin expression in both BT-474 and MDA-MB-231 generated recurrent tumors, as compared to respective vehicle controls. In addition, OC reduced phosphorylation levels of both HER2 and MET in recurrent tumors from BT-474-inoculated animals, with or without neoadjuvant LP treatment. Levels of phosphorylated MET were also reduced modestly in MDA-MB-231 tumors, the significance of which remains to be further elucidated in future studies. These findings are consistent with earlier reports documenting the ability of OC to suppress EMT in BC cells [26]. Earlier studies also validated OC as a MET kinase inhibitor [25,26,31]. Lastly, histopathology of OC-treated recurrent tumors showed evidence of cytolethality, with extensive cell disintegration leaving granular, vacuolar debris and coagulative necrosis. In summary, OC, administered orally, induced potent suppression of tumor recurrence following careful and histopathologically assessed surgical resection of BT-474 generated primary tumors. This finding can be rationalized in light of OC’s inhibition of EMT and HER2/MET-mediated cell invasiveness.

## 4. Materials and Methods

### 4.1. Chemicals and Reagents

All reagents were purchased from Sigma-Aldrich (St. Louis, MO, USA) or VWR International (Suwanee, GA, USA), unless otherwise stated. CA 15-3 (Human) ELISA Kit (catalog Number KA0206) was purchased from Abnova (Walnut, CA, USA). All primary and secondary antibodies were purchased from Cell Signaling Technology (Danvers, MA 01923, USA).

### 4.2. Extraction of (−)-Oleocanthal from Extra-Virgin Olive Oil

OC was extracted from EVOO (The Governor, batch #5-214000-242017). Separation was performed using our novel liquid–liquid extraction technology, extracting EVOO with water followed by ^1^H NMR-guided size exclusion chromatography on Sephadex LH20, using isocratic dichloromethane elution [32]. The identity of OC was unambiguously defined by extensive 1D and 2D NMR analysis using a JEOL Eclipse ECS-400 NMR spectrometer. Pure OC sample (>99% purity by HPLC) was stored frozen in amber glass vials under N_2_ gas [32].

### 4.3. HPLC Analysis

OC purity was assessed using HPLC analysis on a Shimadzu HPLC system equipped with UV/Visible variable wavelength detector. Briefly, OC was dissolved in 100% CH_3_CN. Samples (20 μL) were then injected onto a Phenomenex Cosmosil 5C18-AR-II column (250 × 4.6 mm, 5 μm; Phenomenex Inc., Torrance, CA, USA). The flow rate of the mobile phase (CH_3_CN-H_2_O 1:1, isocratic) was 1.0 mL/min. Detection was by UV absorbance simultaneously at λ=230 and 254 nm, with 13.9 min OC retention time. Data acquisition and analysis were accomplished using Lab Solution™ chromatography software [32].

### 4.4. (−)-Oleocanthal Purity Assessment Using NMR Spectral Analysis

Quantitative ^1^H NMR (q^1^H NMR) and ^13^C NMR analysis were used to assess and confirm OC purity. The OC-rich residue, obtained according to the above-described extraction procedure, was dissolved in 750 μL CDCl_3_ in a 5-mm NMR tube. ^1^H and ^13^C NMR spectra were recorded using tetramethylsilane (TMS) as an internal standard, on a JEOL Eclipse-ECS NMR spectrometer operating at 400 MHz for ^1^H NMR and 100 MHz for ^13^C NMR. A single data set of each of ^1^H and ^13^C-PENDANT NMR experiments confirmed that OC had >99% purity. Typically, 16 scans were collected into 32 K data points over a spectral width of 0−20 ppm with a relaxation delay of 1 s and an acquisition time of 2.1 min for the ^1^H NMR spectrum [32].

### 4.5. In-Vivo Studies

#### 4.5.1. Animals

Female athymic nude mice (Foxn1^nu^/Foxn1^+^, 4–5 weeks old) were purchased from Envigo (Indianapolis, IN, USA). The animals were acclimated to the animal housing facility and maintained under clean room conditions in sterile filter-top cages with Alpha-Dri bedding and housed on high efficiency particulate air-filtered ventilated racks at a temperature of 18–25 °C, with a relative humidity of 55–65% and a 12 h light/dark cycle, for at least one week before the study. The mice had free access to drinking water and pelleted rodent chow (no. 7012, Envigo/Teklad, Madison, WI, USA). Animals were housed in group cages, 10 animals for each experimental group. However, after the surgery, the animals were housed individually for 3–5 days for recovery and wound healing. All animal experiments were approved by the Institutional Animal Care and Use Committee (IACUC), University of Louisiana at Monroe, and were conducted in strict accordance with good animal practice as defined by NIH guidelines (Protocol 16MAR-KES-02).

#### 4.5.2. A Xenograft Model of Tumor Recurrence in Athymic Nude Mice of BT-474 and MDA-MB-231 BC Cells

Foxn1^nu^/Foxn1^+^ nude mice were maintained on sterilized food and water. Ten female nude mice, 4–5 weeks-old, 20–23 g average weight, were used for each group. BT-474 or MDA-MB-231/GFP human BC cells were harvested, pelleted by centrifugation at 850× *g* for 5 min, and re-suspended in sterile serum-free DMEM medium (30–50 μL). Xylazine (1 mL xylazine at 20 mg/mL) was added to 10 mL ketamine at 100 mg/mL to make 11.0 mL at 92 mg/mL of stock. About 1 mL of this solution was diluted with 9 mL sterile normal saline to make a 9.2 mg/mL solution. Cancer cell suspension (5 × 10^6^ cells/50 μL for BT-474 cells and 2 × 10^6^ cells/30 μL for MDA-MB-231 cells) was inoculated subcutaneously into the second mammary gland fat pad just beneath the nipple of each animal after anesthesia to generate orthotopic breast tumors. Primary tumors were surgically excised when they reached an average tumor volume of 400 mm^3^, which was achieved at day 15 post-inoculation for BT-474 tumors and day 21 for MDA-MB-231 tumors. Xenografted mice were anesthetized prior to the surgical excision procedure with ip ketamine/xylazine combination (100 mg/kg/15 mg/kg) [26]. About 15–20 min after administering the anesthetic, animal reflexes were tested by gently tapping the hind legs with a sterile syringe needle, and when animals were fully anesthetized, their primary tumors were surgically excised, and each wound was closed by one or two stitches. Ketoprofen, 1 mg/kg, was used 12 h before and after surgery for effective analgesia. Ophthalmic lubricant was used during the surgery to prevent corneal drying. Bupivacaine (0.25%, 1–2 drops), twice daily, was used topically at the excision wound site to prevent local infiltration along the surgery site during closure, with a maximum dose of 2 mg/kg. The collected primary breast tumors were further stored at −80 °C.

One day after surgical excision of primary breast tumors, animals which originally harbored BT-474 tumors were randomly divided into two groups with five animals in each: (i) vehicle-treated control group and (ii) OC-treated group (10 mg/kg). Similarly, animals which had harbored MDA-MB-231 tumors were randomly divided into two groups, with five animals in each: (i) vehicle-treated control group, and (ii) OC-treated group (10 mg/kg). For both tumor models, treatment was administered orally (p.o.) on a daily basis (7×/week) with vehicle control (water/DMSO) or freshly prepared 10 mg/kg OC. OC was dissolved in the least amount of sterile DMSO (not more than 0.25% *v*/*v*) and the final preparation was given by a ball-tipped plastics gavage needle (70 mm length). The mice were monitored daily for new tumors and body weight, and were carefully observed for general health characters including food/water intake, defecation, urination, and physical activity. In addition, animals were regularly observed to assure that post-surgery wounds were healing and contamination-free. Throughout the experiments, tumor volume (*V*) was calculated by *V* = *L*/2 × *W*^2^, where *L* was the length and *W* was the width of tumors. The results are presented as average ± SD. At the end of the experiments, all mice were sacrificed. Fresh blood samples were collected by using heparinized microtainer tubes and immediately centrifuged at 4 °C at 13,000 rpm for 10 min to prepare serum samples, which were stored at −80 °C until CA 15-3 quantification. Collected tumors and organs were weighed and stored at −80 °C until total protein extraction for Western blot analysis. Percentage of tumor growth inhibition (% TGI) was measured on the last day of the study for drug-treated compared with vehicle-treated mice.

#### 4.5.3. A Xenograft Model of Tumor Recurrence in Athymic Nude Mice of BT-474 BC Cells After LP Neoadjuvant Treatment

All mice were maintained in similar conditions and inoculated as mentioned earlier. After animals developed palpable tumors at an average tumor volume of 30 mm^3^ in BT-474 xenograft, at day-8 LP neoadjuvant treatment (50 mg/kg) was started orally 5×/week. LP was continued until day-19 for a total of two weeks duration. At day-19, after completion of the neoadjuvant treatment, the primary tumors were surgically excised. At day-20, mice were further divided into two groups, (i) vehicle control group (water/DMSO), which stopped dosing of LP treatment (LP→VC), *n* = 5 mice, (ii) OC treatment at 10 mg/kg (LP→OC), daily basis 7×/week, (p.o.), *n* = 5 mice. At the end of the experiments (day 40), all mice were sacrificed. Fresh blood samples were collected by using heparinized microtainer tubes and immediately centrifuged at 4 °C at 13,000 rpm for 10 min to prepare serum, which was then stored at −80 °C until CA 15-3 quantification. Collected tumors and organs were weighed and stored in a similar condition, as mentioned earlier.

All mice were observed and handled carefully in a way similar to above-described methods during surgery and post-surgical treatments.

### 4.6. Quantification of CA 15-3 Serum Levels Using ELISA

All serum samples were prepared from freshly collected blood samples immediately after animals were sacrificed at the end of all experiments. Serum samples were prepared and analyzed, following manufacturer protocols, with the Abnova CA 15-3 (human) ELISA Kit (Catalog Number KA0206). Briefly, 20 µL of serum sample was mixed with 1.0 mL sample diluent. About 200 µL of CA 15-3 standards, diluted specimens, and diluted controls were added into the appropriate wells and gently mixed for 10 s and incubated at 37 °C for 1 h, and then the microtiter plate was rinsed and emptied five times with wash buffer (1×). Enzyme conjugate reagent (200 µL) was dispensed into each well and gently mixed for 10 s followed by incubation at 37 °C for 1 h. In addition, 100 µL of TMB reagent was dispensed into each well and gently mixed for 10 s followed by incubation at room temperature in the dark for 20 min. Finally, the reaction was stopped by adding 100 µL of stopping solution to each well and gently mixing for 30 s. At the end of the experiment, absorbance was read immediately at a wavelength of 450 nm with a microtiter plate reader.

### 4.7. Western Blot Analysis

Breast tumor tissues were stored at −80 °C until protein extraction. At the end of the treatment period, cells were lysed in RIPA buffer (Qiagen Sciences Inc., Valencia, CA, USA) and breast tumor tissues were homogenized in RIPA buffer using an electric homogenizer [26,30]. Protein concentration was determined by the BCA assay (Bio-Rad Laboratories, Hercules, CA, USA). Equivalent amounts of protein were electrophoresed on SDS–polyacrylamide gels. The gels were then electroblotted onto PVDF membranes. These PVDF membranes were then blocked with 2% BSA in 10 mM Tris-HCl containing 50 mM NaCl and 0.1% Tween-20, pH 7.4 (TBST), and then incubated with specific primary antibodies overnight at 4 °C according to the manufacturer protocol. At the end of the incubation period, membranes were washed five times with TBST and then incubated with respective horseradish peroxide-conjugated secondary antibody in 2% BSA in TBST for 1 h at room temperature, followed by rinsing with TBST five times. Blots were then visualized by chemiluminescence according to the manufacturer’s instructions (Pierce, Rockford, IL, USA). Proteins were detected using ChemiDoc XRS chemiluminescent gel imaging system and analyzed using Image Lab software (Bio-Rad Laboratories). The visualization of β-tubulin was used to ensure equal sample loading in each lane. All experiments were repeated three times [26,30].

### 4.8. Hematoxylin and Eosin Y (H&E) Staining

Tumor sample portions were cut into small slices (3–5 mm thick) and then fixed in 10% neutral buffered formalin for 48 h. The tissues were transferred to 70% ethanol, processed, and embedded in paraffin. Paraffin-embedded tumors were sliced into 5 µm sections using a Leica RM2035 microtome, Sections were mounted on positively charged slides, dewaxed in xylene, rinsed in alcohol, rehydrated in water, and finally, tumor slides were stained with H&E. Following a last submersion into xylene, slides were permanently mounted using Permount and a coverslip [31].

### 4.9. Statistical Analysis

Differences among various treatment groups were determined by the unpaired *t*-test using GraphPad Prism Version 8. A difference of * *p* < 0.05, ** *p* < 0.001 was considered statistically significant as compared to the vehicle-treated control group. Percentage of tumor growth inhibition (% TGI) was measured based on tumor volume calculations on the last day of the study for drug-treated compared with vehicle-treated mice. % TGI is calculated as 100 × {1 − [(treated final day − treated day 1)/(control final day − control day 1)]}.

## 5. Conclusions

The current study presents the first evidence for OC potential to inhibit BC locoregional recurrence in luminal HER2^+^/ER^+^ BT-474 tumors. Prevention of tumor recurrence was associated with downregulation of MET and HER2 receptors and suppression of receptor activation. In addition, OC treatment prevented EMT by stabilizing epithelial E-cadherin and reducing the level of mesenchymal vimentin in recurrence tumors, which further reduces the migratory and invasive potential, and would presumably minimize the risk of distant metastases of these tumors. Although this study focused on locoregional and not distant recurrence, current literature correlates locoregional with distant recurrence, especially in patients with lymph-node-positive BC [35,51,52].

Findings from this study have important implications for devising new, clinically useful strategies, and in searching for novel additional approaches, for reducing BC recurrence and improving patient longer-term survival rates. EVOO, which contains the constituent OC at levels (dependent on source and production processing) sufficient to reasonably anticipate health benefits, has been used as food and even remedy throughout human history, prominently, as an epidemiologically identified key component of Mediterranean diets. Other prospective advantages of studying and seeking to develop OC itself include the expected long-term safety profile based on the historical human consumption of olive oil, cost-effectiveness based on the sustained plant supply source, relative ease and economic viability of commercial production, and potential for marketing as a dietary supplement, thus potentially averting the need for full pre-marketing regulatory approval.

## Figures and Tables

**Table 1:** Quantification of serum CA 15-3 levels in BC recurrence models.

CA 15-3	BT-474		MDA-MB-231				BT-474 Recurrence After Neoadjuvant LP Regimen		
	VC	OC	*p*-value	VC	OC	*p*-value	LP→VC	LP→OC	*p*-value
Level (U/mL)	1.9 ± 0.9	0.4 ± 0.4	0.012	2.1 ± 0.8	0.4 ± 0.1	0.002	1.6 ± 1.0	0.4 ± 0.1	0.032

Data presented as mean ± SD. *p*-value represents significance difference compared to respective vehicle-treated control group.

## Supplementary Materials

The following are available online at https://www.mdpi.com/2072-6694/11/5/637/s1, Figure S1: H&E Stained section images of primary and recurrent tumor samples in different study groups, Figure S2: Standard calibration curve used for quantification of animal sera CA 15-3 levels, Table S1: Different organs weights of BT-474 and MDA-MB-231 recurrence mouse models, Table S2: Organ weights of different mice groups in BT-474 LP neoadjuvant therapy recurrence model.
