# Supplementary material for: (−)-Oleocanthal Prevents Breast Cancer Locoregional Recurrence After Primary Tumor Surgical Excision and Neoadjuvant Targeted Therapy in Orthotopic Nude Mouse Models
Source: Cancers (Basel). 2019 May 8;11(5):637. doi: 10.3390/cancers11050637 (PMC6562541; doi:10.3390/cancers11050637)
Supplement: Supplementary file 1 [file cancers-11-00637-s001.pdf]

Supplementary Material

(−)-Oleocanthal Prevents Breast Cancer Locoregional Recurrence After Primary Tumor Surgical Excision and Neoadjuvant Therapies in Orthotopic Nude Mouse Models

Abu Bakar Siddique, Nehad M. Ayoub, Afsana Tajmim, Sharon A. Meyer, Ronald A. Hill and Khalid A. El Sayed

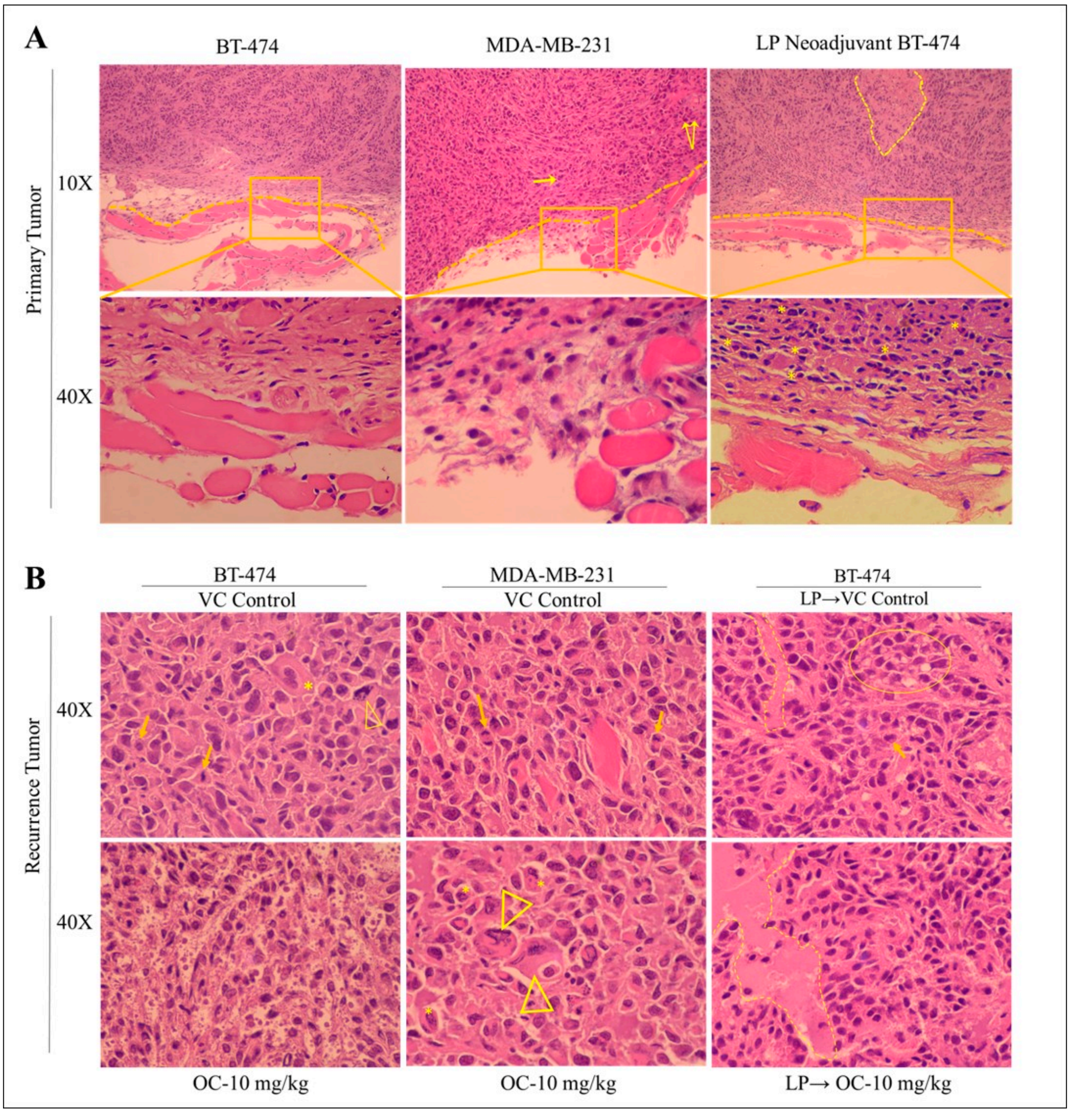

Figure S1. H&E Stained section images of primary and recurrent tumor samples in different study groups. A. Left panel: H&E Stained BT-474 primary tumor. Middle panel: H&E Stained MDA-MB-231 primary tumor. Right panel: H&E Stained BT-474 primary tumor treated with LP neoadjuvant therapy. B. Left panel: H&E Stained BT-474 recurrence tumor. Upper row: Vehicle control-treated tumor. Lower row: OC-10 mg/kg-treated tumor. Middle panel: H&E Stained MDA-MB-231 recurrent tumor. Upper row: Vehicle control-treated tumor. Lower row: OC-10 mg/kg-treated tumor. Right panel: H&E Stained BT-474 recurrent tumor treated with LP neoadjuvant therapy. Upper row: LP→ Vehicle control-treated tumor. Lower row: LP→OC-10 mg/kg-treated tumor. Arrows indicate mitotic figures. * indicate a binucleated cell. Dotted line indicate fibrotic tracks. Circle indicate vacuolization.

All excised primary tumors show a loose connective tissue capsule around the margin. Some include regions of host muscle adjacent to the capsule of the excised tumor. BT-474 cells tumor differs from that of MDA-MB-231 cells tumor in exhibiting greater fibrosis. BT-474 tumor fibrosis appeared unaffected by lapatinib. Occasional mitotic figures (arrows) are seen in low magnification micrograph of the MDA-MB-231 tumor. The low magnification photomicrograph of lapatinib-treated BT-474 tumor illustrates an encircled region of coagulative necrosis. Intratumor eosinophils with multilobular nuclei are indicated by the * in the higher magnification micrograph.

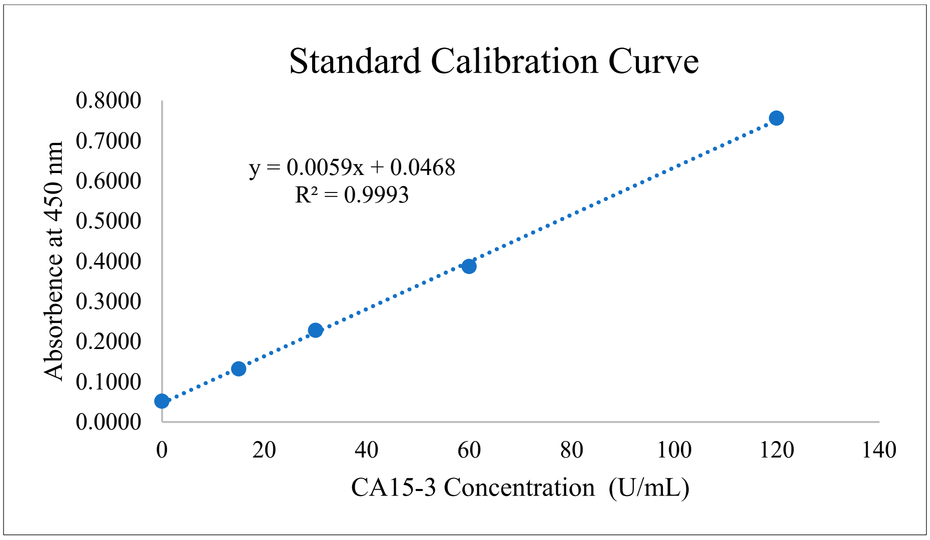

Figure S2. Standard calibration curve used for quantification of animal sera CA 15-3 levels.

Table S1. Different organs weights of BT-474 and MDA-MB-231 recurrence mouse models.

Organ/Group	BT-474			MDA-MB-231		
	VC	OC	*p*-Value	VC	OC	*p*-Value
Brain (g)	0.37 ± 0.02	0.43 ± 0.06	ns	0.46 ± 0.11	0.50 ± 0.06	ns
Heart (g)	0.12 ± 0.02	0.13 ± 0.03	ns	0.17 ± 0.05	0.19 ± 0.06	ns
Lung (g)	0.15 ± 0.00	0.23 ± 0.06	ns	0.29 ± 0.11	0.32 ± 0.04	ns
Liver (g)	1.09 ± 0.23	1.24 ± 0.22	ns	1.30 ± 0.25	1.53 ± 0.18	ns
Kidney (g)	0.27 ± 0.01	0.36 ± 0.03	ns	0.39 ± 0.04	0.40 ± 0.05	ns
Spleen (g)	0.17 ± 0.00	0.29 ± 0.21	ns	0.24 ± 0.09	0.25 ± 0.06	ns

Data are means ± SD. ns = indicating statically not significant.

Table S2. Organ weights of different mice groups in BT-474 LP neoadjuvant therapy recurrence model.

Organ/Group	BT-474		
	LP→VC	LP→OC	*p*-Value
Brain (g)	0.44 ± 0.06	0.46 ± 0.04	ns
Heart (g)	0.15 ± 0.07	0.18 ± 0.04	ns
Lung (g)	0.30 ± 0.08	0.22 ± 0.06	ns
Liver (g)	1.08 ± 0.22	1.20 ± 0.04	ns
Kidney (g)	0.33 ± 0.08	0.36 ± 0.04	ns
Spleen (g)	0.08 ± 0.01	0.15 ± 0.06	ns

Data are means ± SD. ns = indicating statically not significant.

© 2019 by the authors. Licensee MDPI, Basel, Switzerland. This article is an open access article distributed under the terms and conditions of the Creative Commons Attribution (CC BY) license (http://creativecommons.org/licenses/by/4.0/).
